# Mitochondrial Dynamics in Basal and Stressful Conditions

**DOI:** 10.3390/ijms19020564

**Published:** 2018-02-13

**Authors:** Naima Zemirli, Etienne Morel, Diana Molino

**Affiliations:** 1 Institut Necker-Enfants Malades (INEM), INSERM U1151-CNRS UMR 8253, Paris F-75014, France; naima.zemirli@inserm.fr (N.Z.); etienne.morel@inserm.fr (E.M.); 2 Université Paris Descartes-Sorbonne Paris Cité, Paris F-75993, France

**Keywords:** mitochondrial dynamics, autophagy, membranes

## Abstract

The historical role of mitochondria resides in converting the energy released during the oxidation of macromolecules (carbohydrates, lipids and proteins) into adenosine tri-phosphate, a major form of chemically stored energy which sustains cell growth and homeostasis. Beyond this role in bioenergetics regulation, mitochondria play a role in several other cellular processes including lipid metabolism, cellular calcium homeostasis, autophagy and immune responses. Furthermore, mitochondria are highly dynamic organelles: as all other cellular endomembranes, they are continuously moving along cytoskeleton, and, most importantly, they constantly interact one with each other by membrane tethering, fusion and fission. This review aims to highlight the tight correlation between the morphodynamics of mitochondria and their biological function(s), in physiological as well as stress conditions, in particular nutrient deprivation, pathogen attack and some human diseases. Finally, we emphasize some crosstalk between the fusion/fission machinery and the autophagy pathway to ending on some speculative hypothesis to inspire future research in the field.

## 1. Introduction

Mitochondria are the powerhouses of the cell, generating a large part of cellular ATP content (adenosine tri-phosphate). These organelles are also involved in vital metabolic processes, such as calcium and iron homeostasis, redox signalling, programmed cell death, innate immunity and finally participate in the regulation of various physiological processes [1]. 

In the past century, advances in live cell imaging revealed the dynamic behaviour of mitochondria (MT) and MT network. Indeed, to maintain their homeostasis, these organelles form elongated tubules that continually divide and fuse. This morphology results from a balance between two opposite processes: fusion and fission. When this equilibrium is broken, mitochondria lose their characteristic shape. Hence, if MT fusion is reduced, the mitochondria become fragmented because of unbalanced fission. Conversely, if MT fission is reduced, the mitochondria become elongated and excessively interconnected because of unbalanced fusion.

The molecular machinery that mediates this organelle fission and fusion is thus necessary to maintain MT integrity and is tightly regulated by post-translational modifications and protein-protein interactions [2]. 

**Figure 1:**
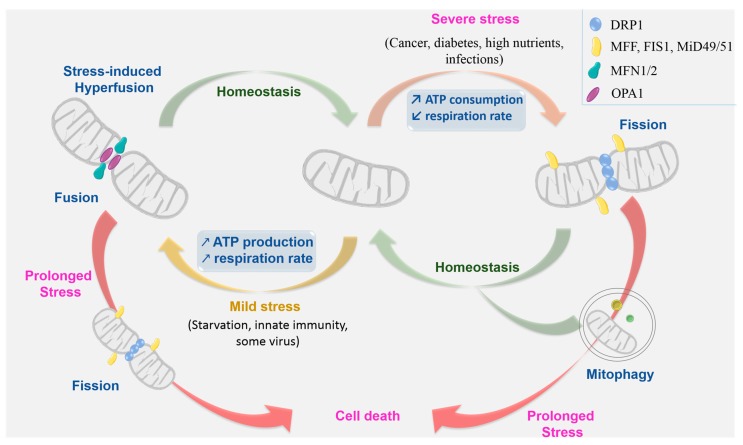
Mitochondrial dynamics: machinery and context. Mitochondria continuously undergo two membrane related opposite processes: fission and fusion. According to cell context and circumstances, the equilibrium between fission and fusion could be altered and the balance leans towards one of these two processes. When cells are subjected to mild stresses, mitochondria form an elongated and interconnected network, they resist mitophagy and increase ATP production to adjust to cellular stresses as nutrient deprivation. Conversely, in the case of severe stresses, mitochondria present a fragmented form. Failure of any of these pathways to maintain homeostasis, results in elimination of the entire mitochondria via mitophagy, or, if stress is prolonged, via apoptosis. DRP1: Dynamin-Related Protein 1; MFF: Mitochondrial Fission Factor; FIS1: FISsion 1; MiD49/51: MItochondrial Dynamics 49/51; Mfn1/2: Mitofusin 1/2; OPA1: OPtic Atrophy 1.

In mammals, MT-fusion events are mediated by mitochondrial proteins belonging to the family of -related GTPases: Mitofusin 1 (MFN1), Mitofusin 2 (MFN2) and OPtic Atrophy 1 (OPA1). As mitochondrion is a double-membrane organelle, this fusion occurs in a two-step process: MFN1 and MFN 2 ensure first the fusion of the outer mitochondrial membranes (MOM) [3] while OPA1 mediates the fusion of the inner mitochondrial membranes (MIM). These two phases are coordinated and occur almost simultaneously (Figure 1).

Mitofusins were first described to be required for MT fusion in drosophila [4] and yeast [5]. In mammals, mouse knockout models illustrate their importance in fusion. Indeed, deficient mouse embryonic fibroblasts (MEFs) in either Mfn1 or Mfn2 present highly fragmented mitochondria compared to the tubular and interconnected mitochondrial network observed in wild-type (WT) cells [3]. Beside its role in MT fusion, MFN 2 is also implicated in the formation of Endoplasmic Reticulum (ER)-MT contact sites [6] and in MT-lipid droplets interaction [7]. 

The mechanism by which mitofusins mediate MT fusion is not fully understood, although it was suggested that MFN 1 and MFN 2 form homo- or hetero-oligomeric complexes by interacting in trans between neighbouring mitochondria, thereby promoting their tethering and subsequent fusion of MOM [8]. 

OPA1, whose gene mutation is associated with a dominant optic atrophy disease [9] (Table 1), is localized to the MIM and the MT intermembrane space. *Opa1*^−/−^ cells present fragmented mitochondrial morphology, although some MOM fusion events were described [10]. Moreover, Opa1 depletion triggers other cellular defects including reduction and disorganization of cristae membranes, reduced respiration capacity, altered mtDNA maintenance and increased sensitivity to apoptosis [11]. Different isoforms of OPA1 arise through differential RNA splicing and protein processing. Importantly, it has been shown that MT fusion depends on a proper ratio between two major forms of OPA1: The Long (L-OPA1) and Short (S-OPA1) isoforms. In fact, L-OPA1 is associated with efficient MT fusion, while S-OPA1 is thought to be a fission mediator [12]. 

OPA1 processing is catalysed by the ATP-dependent protease YME1L and the membrane potential-dependent protease OMA1 (metallopeptidase Overlapping with the m-AAA protease1). Thus, proteolysis via Yme1L is responsible for oxidative phosphorylation-dependent stimulation of inner-membrane fusion. In contrast, the dissipation of membrane potential triggers OMA1 activation and subsequent cleavage and inactivation of OPA1 [13]. Various cellular stresses can activate Oma1 to cleave Opa1 [14]. Cells in which Oma1 expression is down-regulated recovered fused mitochondria more quickly than control cells after CCCP-treatment (CarbonylCyanure m-ChloroPhénylhydrazone) and became less sensitive to staurosporine-induced apoptosis [15], suggesting that, in these conditions, OMA1 regulates the functions of the dynamin by modulating the abundance of the L-isoform [15]. This mechanism likely contributes to the mitochondrial fragmentation observed in different forms of mitochondrial dysfunction [16].

In addition to proteolytic regulation of OPA1, some post-translational modifications have also been described. In neonatal cardiac myocytes, high-glucose exposure triggers increased O-GlcNAcylation of OPA1, leading to mitochondrial dysfunction by increasing mitochondrial fragmentation [17]. Moreover, prolonged treatment of rat kidney proximal tubular cells by prostaglandin showed that OPA1 is subjected to ubiquitination and subsequent degradation [18]. In the context of heart pathological stress, OPA1 is hyperacetylated and inactivated [19]. It has been shown that the mitochondrial deacetylase SIRT3 was capable of deacetylating OPA1 and elevating its GTPase activity. SIRT3-dependent activation of OPA1 contributes to the preservation of mitochondrial networking and protection of cardiomyocytes from doxorubicin-mediated cell death [19].

On the other hand, genetic and cell biology studies identified two classes of proteins required for MT fission process. In mammals, the GTPase dynamin-related protein 1 (DRP1) [20], is described to be the major effector of this process. Considering the cytosolic localization of this protein, its recruitment to the mitochondrial surface requires specific adaptor proteins on the MOM (Figure 1). So far, this second class of proteins includes Fis1, Mff, MiD49 and MiD51 (FISsion 1 protein, Mitochondrial Fission Factor, and Mitochondrial Dynamics protein 49/51; Figure 1) [21].

Inhibition of Drp1 function, either by RNA interference or by expressing a dominant-negative form of the protein, results in defective MT fission and the occurrence of a hyperfused mitochondrial network [20]. During fission, DRP1 is recruited on mitochondrial tubules and assembles into oligomeric spirals that mediate constriction and scission of the mitochondrion. Recently, Voeltz and colleagues [22] have demonstrated that a dynamin family member “Dyn2” is required for scission process. Indeed, Dyn2-depleted cells displayed a hyperfused mitochondrial network that was similar to mitochondria in Drp1-depleted cells. The authors proposed that Drp1-mediated constriction facilitates Dyn2 assembly, which induces membrane fission to complete division [22].

More investigation in fission mechanism allowed to highlight the involvement of MT-ER-Actin nexus in the induction of initial membrane curvature on mitochondria to mark specific sites for the recruitment of DRP1 and hence trigger fission process [23,24].

The regulation of DRP1 properties, like GTPase activity, mitochondrial translocation and assembly is critical. Thus, regulation of Drp1 and/or its partners by post-translational modifications is important for DRP1 cycling between the cytosol and mitochondria. One of the most studied post translational modifications of DRP1 is phosphorylation, which occurs at several sites and is regulated by various kinases and phosphatases. In general, DRP1 phosphorylation at Ser637 inhibits its activity, while phosphorylation at Ser616 is mostly associated with DRP1 activation.

cAMP-dependent protein kinase A (PKA) phosphorylates Ser637 of human DRP1 and this modification inhibits mitochondrial fission through the inhibition of GTPase activity and eventually mitochondrial recruitment of DRP1 [25], as during cardiac ischemic injury model [26] and starvation (as detailed later). A kinase anchoring protein 1(AKAP1, also known as Rab32), is a scaffold protein localized on the MOM and involved in the mitochondrial recruitment of DRP1. The PKA/AKAP1 complex regulates the phosphorylation of DRP1 on Ser637 to preserve mitochondrial network integrity and cell survival [27]. Calcineurin dephosphorylates Drp1-Ser637(P) and stimulates DRP1 translocation to the mitochondria [28]. Surprisingly, phosphorylation at the same residue could have opposite effect on DRP1 activity and mitochondrial morphology: the voltage-dependant Ca^2+^ channels (VDCCs)-associated Ca^2+^ signalling stimulates DRP1 phosphorylation at Ser637 via the activation of Ca^2+^/calmodulin-dependent protein kinase Iα (CaMKIα). In neurons and HeLa cells, this phosphorylation is associated with an increase of DRP1 translocation to mitochondria and thus fission process [29]. On the other hand, during mitosis, the kinase activity of CDK1/cyclin B is stimulated at the mitochondrial membranes and mediates the phosphorylation of human DRP1 on Ser616, leading to its oligomerization and subsequent mitochondrial fission [30]. In neurons, protein kinase Cδ (PKCδ) catalyses the phosphorylation of DRP1 Ser616 leading to mitochondrial fragmentation and impaired mitochondrial function under oxidative stress conditions [31]. During the process of reprogramming to induced pluripotent stem (iPS) cells, somatic cells switch from oxidative to glycolytic metabolism. It has been demonstrated that the phosphorylation of DRP1 at Ser616 by ERK1/2 is an early and necessary step in this process [32]. Other phosphorylation sites have also been described, for example, DRP1-Ser693 which is targeted by GSK3β, leading to the inhibition of DRP1 GTPase activity and the formation of an elongated mitochondrial network in the context of oxidative stress [33].

DRP1 is also subjected to other post-translational modifications. In the context of Alzheimer’s disease, β-amyloid protein stimulates NO production to cause *S*-nitrosylation of Drp1 at Cys644. This modification is associated with enhanced GTPase activity and Drp1 oligomerization, leading to excessive mitochondrial fission in neurons, which causes synaptic loss and neuronal damage in the brains of Alzheimer’s disease patients [34]. 

DRP1 is also targeted by ubiquitination. This modification is mainly catalysed by MARCH5, a mitochondrial E3 ligase which ubiquitinates DRP1 on the MOM. The effect of MARCH5 is debated as its knockdown or the overexpression of the mutant form lacking ubiquitin ligase activity, showed opposed effect on mitochondrial morphology [35,36]. 

In addition to ubiquitination, DRP1 is also regulated by SUMOylation. Overexpression of SUMO1 (Small ubiquitin-related modifier 1induces mitochondrial fission via the SUMOylation of DRP1 in a Bax/Bak dependent manner during early apoptosis progression [37]. MAPL, a mitochondrial SUMO E3 ligase has also been described as DRP1 regulator and its overexpression stimulates mitochondrial fragmentation [38].

Finally, *O*-GlucNAcetylation of DRP1 at Thr585 and Thr586 in rat neonatal cardiac myocytes has been described. This modification is associated with increased levels of GTP-bound active DRP1, its mitochondrial translocation and the subsequent induction of mitochondrial fission [39].

A defective mitochondrial dynamic machinery could have important physiological consequences including embryonic lethality and diseases [2] (Table 1), suggesting that fission and fusion go far beyond the morphological aspect of mitochondria. Mitochondrial dynamics have been linked to multiple mitochondrial functions. Thus, fusion process allows contents exchange, such as mtDNA, proteins, lipids and metabolites between mitochondria, all necessary for maintaining genetic and biochemical homogeneity within the mitochondrial population. The fusion process is also well known to be important for oxidative phosphorylation activity, particularly through the regulation of mtDNA levels. Fission, on the other hand, ensures the quality control of mitochondria by facilitating the removal of damaged organelles via mitophagy, facilitates their transport along the cytoskeletal network and is essential for separating mitochondria into two daughter cells during cell division [40,41]. 

In this review, we emphasize on the profound relationship between MT fusion/fission dynamics and stress conditions, in particular in relation with nutritional stress and pathogen attack. Several studies highlighted the MT dynamics modulation in response to certain types of stress. Depending on stress type and severity, mitochondria adapt their form, either by promoting a fragmented or a fused shape. These changes are part of the cellular adaptation to maintain cell homeostasis and regulate cell survival [42]. Because of the crucial importance of MT fusion and fission, even mild defects in this equilibrium could be associated with diseases. We will thus give an overview of mitochondrial dynamics-associated pathologies to finally conclude with some provocative hypothesis on the bidirectional connection between the autophagic machinery and MT morphodynamics.

## 2. Mitochondrial Fusion Maintain Metabolic Homeostasis during Nutritional Stress

Under stressful conditions, the cell implements protective mechanisms in the aim to maintain its homeostasis. The established stress response(s) results either in a positive event which opposes the environmental attack, or, in the case of failure, in cell death.

In this context and in various cell models, some selective mild stresses including UV irradiation, inhibition of RNA transcription, inhibition of protein translation and moderate nutrient starvation, trigger the so called “SIMH” response, for “Stress-Induced Mitochondrial Hyperfusion response”. The SIMH requires the fusion machinery proteins MFN 1 and OPA1, as well as the MIM protein SLP2 (Stomatin-Like Protein 2). SIMH is thought to be a pro-survival response to stress, since it is associated with increased ATP production and NF-κB transcription factor activation [43,44].

During starvation, cell survival is mainly dependent on a catabolic pathway called autophagy, which consists on a self-eating process resulting on a degradation of cytoplasmic components to provide energy to cells. Nevertheless, two studies elegantly demonstrate that following starvation, the resulting MT network spared from degradation [45,46]. Interestingly, starved Opa1-deficient cells are degraded by autophagy, suggesting that MT fusion during nutrient depletion protects them from autophagic degradation [46]. Furthermore, Guedouari and co-authors have recently shown that starvation-induced mitochondrial hyperfusion also depends upon the energy sensor Sirtuin 5 (Sirt5) [47]. Indeed, deletion of Sirt5 induces DRP1 accumulation, mitochondria fragmentation and thus their degradation via autophagy. In summary, while unhealthy mitochondria appear fragmented and are eliminated via mitophagy, a selective form of autophagy [48], mitochondria become tubular and form extensive and interconnected networks under starvation and are thus protected from autophagic degradation. Altogether these data suggest that the modulation of MT fission/fusion machinery serves to maintain metabolic homeostasis during stress conditions. Indeed, in stress conditions, cells need to maintain their most competent means for ATP production. In yeast, non-fermentable culture conditions that force increased Oxydative phosphorylation activity are accompanied by elongation of the mitochondrial network [49,50]. This result has also been confirmed in human cells models suggesting that efficiency of oxidative phosphorylation is associated with mitochondrial connectivity; hence highly interconnected network correlates with increased ATP production under different conditions, such as tumour regression, cell cycle progression [51,52], as well as different stress stimuli including UV-C radiation, actinomycin D and cycloheximide [43,45].

Mechanistically, if the main actors of the fusion machinery (OPA1, MFN 1 and to a lesser degree MFN 2) are essential for the starvation-induced mitochondrial elongation, these proteins does not present any obvious modifications justifying the change of mitochondrial shape. Conversely, it has been suggested that MT elongation is mediated via a defect in fission machinery. Indeed, Chang and collaborators showed that the fission protein DRP1 is inhibited through its phosphorylation at Ser637 residue, close to its GTPase domain, this phosphorylation is mainly catalysed by protein kinase A (PKA), which responds to modulation in cAMP/ATP ratio [25]. In the same line, AMPK activation and subsequent mTOR (Mammalian/Mechanistic Target of Rapamycin) inhibition—two major energy and nutrient sensors—have been also shown to induce MT fusion [53]. Thus, phosphorylated DRP1 is sequestered in the cytosol, allowing unopposed fusion process. 

Dephosphorylation of DRP1 at Ser637 and its subsequent activation is achieved by the Ca^2+^-dependent phosphatase calcineurin and triggers MT fission [28]. Metabolic stimuli associated with calcium levels modulation are thus relayed into alterations in MT general morphodynamics. For example, when Ca^2+^-buffering activity is altered in mitochondria, the cytosolic calcium level will increase and this eventually triggers calcineurin-mediated MT fission. Moreover, calcineurin deficient skeletal muscles in mice exhibit a highly elongated mitochondrial network and increased respiratory chain activity [54].

Otherwise, in the case of severe damages as for example in the case of prolonged nutrient stress, mitochondria are degraded by mitophagy or apoptosis. Before undergoing this fate, mitochondrial fission machinery is activated, leading to the facilitation of these two processes [55,56]. Mitophagy is a quality control process by which mitochondria are selectively degraded by the autophagosomal pathway. The PINK1 kinase and the Parkin E3 ubiquitin ligase are highly solicited during this process. PINK1 accumulates on the defective mitochondria and recruits Parkin to the MT surface [57]. Parkin catalyses the degradative ubiquitination of many proteins in the MOM including MFN 1, MFN 2 and two mitochondrial proteins involved in organelles trafficking called Miro1 and Miro2 [56]. This degradation step is thought to isolate defective organelles from the mitochondrial population. DRP1 is then recruited to MT surface where it catalyses fission process.

Finally, in response to energy-related stress, AMPK plays a critical role in the activation of fission machinery by mediating the phosphorylation and the subsequent activation of the MT receptor of DRP1, MFF [58]. Similarly, another DRP1 receptor, named MiD51, appears to be implicated in stress-induced mitochondrial fission. Indeed, this receptor acts as a sensor for the dinucleotides ADP and GDP levels thanks to its high-affinity binding sites. This binding seems to be essential for the activation of MiD51 [59]. In this case, it is possible that MT fission aims to reduce the size of mitochondria, so that they are more suitable to be engulfed by autophagosome.

## 3. Mitochondria Fusion Governs Innate Immunity

Beside their role in cell metabolism, mitochondria and mitochondria dynamics have been more recently involved in innate immunity. Upon viral entry, two independent pathways induce cell responses which ultimate in pro-inflammatory cytokines production. The first one, at endosomes, where Toll-like receptor 3 recognizes dsRNA and the second one, starts in the cytoplasm and rapidly involves the mitochondrial membranes and machineries. Briefly either one of the two RNA helicases RIG1 and MDA-5 (respectively retinoic acid-inducible gene I and melanoma differentiation-associated gene 5) detect and bind cytoplasmic viral dsRNA and then translocate to the MOM to interact with MAVS (mitochondrial antiviral signalling) protein [60]. Such interaction is essential for a proper immune response, since it was shown that MAVS deletion abolishes the production of type I interferons (IFNs) and inflammatory cytokines, emphasizing the importance of the mitochondria in antiviral innate immunity [61,62,63]. MAVS functioning is strictly associated to both MT potential and morphology. For instance, CCCP treatment—a well-known protonophore that dissipates MT potential and induces MT fragmentation—blocks the antiviral immune responses as well [64]. Also, *Mfn1*^−/−^ and *Mfn2*^−/−^ derived MEFs display fragmented MT and are impaired for the production of IFNs and other proinflammatory cytokines in response to viral infection [65]. MAVS seems to localize at MOM but also to other MT subdomains, such as MAMs [66] which are MT membrane regions close associated to the ER and enriched in cholesterol and neutral lipids. Furthermore, MAVS also localizes to other intracellular membranes, peroxisomes for instance [67]. The nature of these different subcellular localizations is unclear but has been suggested to relate to MAVS’ function(s) regulation (repression/induction). 

DsRNAs (expected to mimic viral RNAs) have been shown to promote mitochondrial elongation and furthermore modulation of host Drp1 and Mfns differentially modulates antiviral signalling pathway [68]. For instance, HIV (Human Immunodeficiency Virus) induces Mfn1 expression and reduces Drp1 expression promoting so an elongated MT network in infected cells. A similar elongated MT network is induced by Dengue and SARS (Severe Acute Respiratory Syndrome) viruses, via inhibition of Drp1 dependent MT fission [69,70]. Such elongated mitochondrial network from infected cells promotes viral replication, increase cell respiration, inhibits apoptosis and alter MAMs integrity with consequent inhibition of the immune signalling and interferon production [70,71]. Shi and colleagues [72] have shown that the SARS virus protein ORF-9b bind and induces proteasomal degradation of both DRP1 and MAVS causing both MT fusion and disruption of MAVS downstream signalling, resulting in reduction of the IFN production. However, another study suggest that Dengue virus suppresses mitochondrial fusion via MFNs inhibition [72]. Yu and colleagues [72] revealed that MFN1 is also required in antiviral signalling pathway and a dominant-negative MFN1 mutant which blocks mitochondrial fusion, also reduces antiviral signalling and enhances Dengue infection. 

Some other viruses, such us Hepatitis B and C virus (HBV and HCV) New Castle Disease virus (NDV) and measles viruses, induce instead mitochondrial fission and mitophagy which also favour viral replication [73,74,75,76]. Also the Influenza A (subtype H1N1) full-length PB1-F2 protein targets MT and causes loss of mitochondrial membrane potential and mitochondrial fragmentation [77]. In contrast, a PB1-F2 variant (C-terminal truncated form) does not alter mitochondrial membrane potential nor induces fragmentation. Classical swine fever virus (CSFV) and HCV impact similarly MT network (increased fission). Inhibition of mitochondrial fission by silencing Drp1 during HCV infection enhanced IFN production indicating that HCV-mediated altered mitochondrial dynamics serves to perturb the antiviral defence [74]. Both CSFV and HCV also stimulates Parkin and PINK1 expression leading to increased mitophagy [75,78]. The molecular mechanism of how HCV increases Parkin/PINK1 associated mitophagy remains unclear, even if a yeast-two-hybrid assays and immunoprecipitation experiments suggest that the HCV core protein binds to Parkin [79]. Interestingly enough inhibiting MT fission and mitophagy induced by HCV, induces apoptosis [73].

As already mentioned, cells with an elongated MT network display higher resistance to apoptosis. However a fragmented MT network is not always associated with increased apoptosis but on the contrary can also protect cells from apoptotic death [80]. For instance, in HCV-infected cells (with fragmented mitochondria induced by the infection) there is no apoptosis. Furthermore, in infected cell, reduction of mitochondrial fission increased apoptosis [74] suggesting that MT fission in the context of HCV infection rather promotes cell survival. A similar strategy of perturbation of mitochondrial dynamics and attenuation of apoptosis is also used by hepatitis B virus [73]. Interestingly enough, Kim and colleagues also showed that mitochondrial fission correlates with inhibition of HCV secretion. The precise molecular mechanisms underlying the role of MT dynamics in HCV secretion still remain to be elucidated, however inhibition of mitochondrial fission also led to a decline in glycolysis and total cellular ATP levels and accumulation of ROS: such a cellular stress situation may induce damage in endomembranes stability and functions, which are essential for HCV secretion. 

From all these studies stems out that viruses may modulates MT dynamics and antiviral responses in different (even opposite) way, given the role of mitochondria in innate immune signalling, it is licit but probably too simplistic, to speculate that virus-induced disruption of mitochondrial dynamics is just an evolutionary acquired strategy to impair host innate immune signalling.

Apart from being a platform for early cell responses to viral entry, MT and MT dynamics play also roles in other kind of infection and more generally in immune responses. As already mentioned MT dynamics are regulated by several MT membrane-bound GTPases, such as Rho GTPases (MIRO1 and MIRO2), mitofusins (MFN1 and MFN2), DRP1 and OPA1 [81,82]. Upon Cholera infection, the cellular effector protein VOPE (for *Vibrio cholerae* Type 3 secretion system effector) was shown to localize to mitochondria and interferes with the function of mitochondrial Rho GTPases MIRO1 and MIRO2 [83]. VOPE co-localizes with DRP1 positive fission sites in transfected CHO cells and impairs MFN1-induced MT fusion suggesting that VOPE modulates MT dynamics including fusion and fission by interacting with the MIRO GTPases. All together these studies suggest that MT shape and fusion/fission machinery are tightly associated to their role in responding to pathogen attack, either viral or bacterial. 

Upon infection or tissue damage the multiprotein complex known as inflammasome activates an inflammatory cascade which activates caspase-1 and induces secretion of interleukin-1β or -18 to provide host innate immune protection. Beside the fact that inflammasome activation is a key aspect of the innate immunity, several metabolic disorders as well as Alzheimer’s disease have been associated to inflammasome deregulation [84], reason why great efforts in understanding basic mechanisms of inflammasome have been made in recent years. In particular, a member of one kind of inflammasome, NLRP3 has been associated to MT dynamics. Indeed RNA virus infection induces the formation of the NLRP3-MFN2-MAVS complex, which is required for activation of NLRP3 inflammasome, suggesting that MT fusion is favourable to NLRP3 inflammasome assembly [85]. On the other hand, Wang and collaborators showed that RNA viral infection promotes DRP1 phosphorylation, followed by DRP1-dependent MT fission and subsequent activation of the NLRP3 inflammasome [86].

Finally, a third study proposed that defect in MT fission results in the activation of the NLRP3 inflammasome in LPS/ATP and LPS/Nigericin stimulation context [87]. Thus, authors suggested that decreased expression of the fission protein Drp1 causes MT elongation and this MT conformation somehow creates a favourable context for NLRP3 inflammasome activation in an ERK-dependent manner, thereby leading to diseases associated with deregulated inflammation.

From all these studies, it appears that the MT fusion/fission machinery is tightly connected to the MT role in cell immune responses and this opens important perspectives for new drug discoveries against pathogens.

## 4. Genetic Mutations in Mitochondrial Fusion/Fission Machinery Led to Pathology

Mutations in genes encoding proteins involved in MT fusion/fission machinery land severe pathologies, especially correlated with muscles, brain and nervous system symptoms. This is expected considering that both Drp1 and Mfn2 are essential for proper brain development [80,88,89]. Some human pathologies that have been associated with different mutations in the same gene are summarized in Table 1. It is clearly established that mutations in fusion/fission machinery cause disorders and, vice versa, several disorders such as neurodegenerative diseases and metabolic disorders such as type 2 diabetes are associated with dys-regulation of MT fusion/fission complexes. A putative link between neurodegenerative disorders and Drp1 has been found. For example, some forms of Parkinson disease are caused by mutation in either Parkin or Pink1 and cause aberrant accumulations of DRP1 in neuron which associate with increased MT fission, oxidative stress and reduced ATP production in neurons [90,91]. 

PINK1 and Parkin, as already described, target damaged mitochondria for elimination via mitophagy [92]. More recently, both proteins have been described to participate in a new vesicular pathway consisting in mitochondrial-derived vesicles involved in mitochondrial antigen presentation [93]. This novel endolysosomal pathway is specifically inhibited by PINK1- and Parkin-dependent mitophagy and is independent from autophagy, which on the contrary was shown to enhance antigen presentation [94]. This new discovery by Mathheoud and co-authors not only represents an important step forward in understanding the aetiology of Parkinson Disease but also highlights a new link between MT dynamics and autoimmune mechanisms.

Another neurodegenerative disorder characterized by defect in MT dynamics is Alzheimer’s disease. Fibroblasts from patients show elongated mitochondria associated with reduced DRP1 expression levels [95]. In addition, the same study indicates that β-amyloid accumulation triggers this reduction in Drp1 expression. Finally, the mutant form of huntingtin protein, involved in Huntington disease, interacts with DRP1 increasing MT fission in fibroblasts derived from patients [96]. 

If in one side it is clear that Drp1 defect mainly associates with brain disorders, on the other hands, Mfn2 mutations rather cause metabolic disorders, such as diabetes and obesity. Indeed, MT size is reduced in the skeletal muscle of obese and type 2 diabetic patients, in association with altered expression level of Mfn2 [97,98]. It is also known that Mfn2 loss of function alters cell respiration and MT potential [99], suggesting that the link between MT fusion and diabetic condition may pass by loss of proper MT respiration and glucose consumption and thus possibly associates with insulin resistance.

Physiologically, MT network appears greatly fragmented during mitosis to assure equal distribution in daughter cells upon cell division. Furthermore, during asymmetric cell division MT are inherited unequally. For instance, stem cells can divide asymmetrically to generate a new stem cell (mother) and a progenitor cell (daughter). This latest will eventually undergo to differentiation process, essential for organogenesis and thus development but also for cancer aggressiveness. Daughter cell acquires healthier MT, while stem-like mother cell keeps the aged and damaged ones. Inhibition of mitochondrial fission disrupted mitochondria segregation and caused loss of stemless traits in the progeny, suggesting the essential role of fission components also in asymmetric cell division [100]. Interestingly DRP1 has been suggested to directly interact with apoptosis related proteins to block MT fission, leading to long interconnected organelles as well as a delay in cell death [101,102].

MT network has been found altered in different kind of cancer, which is not surprisingly, considering that MT dynamics are essential for cell division and cell death, process that appear defective in cancerous cells. In general, the majority of studies that have explored MT morphology in tumour cells suggest a great association of cancerous state with MT fission [103]. Enhanced fission or reduced fusion are linked to several cancer-related phenotypes including cell cycle progression and resistance to apoptosis; conversely Drp1 inhibition or Mfn2 overexpression promotes cell cycle arrest [104] and increases spontaneous apoptosis [105]. Mitochondrial fragmentation also enhances glycolysis, limits ROS accumulation and also protect cancer cells from cytotoxic therapies [106]. Interestingly, while the mitochondrial network appear fragmented in cancer cells, changes in glucose availability [52], as well as treatment with anticancer drugs such as MAPK- or PI3K-inhibitors induced an elongated MT network [107,108,109]. 

Thus, the balance of fission/fusion into the MT network may play a key role in the control of cell fate and targeting of MT dynamics it is an interesting target for anti-cancer therapy.

## 5. Concluding Remarks: Is Autophagy Key for a Better Understanding?

The maintenance of a functional MT networking is crucial for overall cell health and homeostasis. Unhealthy MT are mostly degraded by mitophagy: in response to stimuli that induce mitophagy, certain MT fragment and these are so “eaten.” Conversely, during macroautophagy (induced by several stressful conditions), mitochondria hyperfuse to escape autophagosome engulfment and so sustain cell survival.

It has been suggested that MT dynamics may function as a quality control mechanism that governs mitochondrial turnover [119]. To sustain this hypothesis, authors demonstrate that fusion triggers fission and this cycle of fusion/fission segregates less active mitochondria for degradation via mitophagy. Indeed, inhibiting the occurrence of cycles of fusion and fission by Fis1 RNAi or the overexpression of a dominant negative form of Drp1 causes accumulation of damaged mitochondrial material via inhibition of autophagy. DRP1-dependent fission goes along with increased respiration and ROS production in high glucose conditions, a condition that may ultimate in apoptosis when long lasting [120]. Besides, OPA1 processing is induced by the dissipation of MT membrane potential [16]. These observations overall suggest that MT fusion is a protective and necessary state to promote MT functioning in bioenergetics, while fragmentation rather associates with stressful states (Figure 1). Despite the several studies describing the important function(s) of MT dynamics in cellular stress situations, it is important to highlight the fact that most of these studies were conducted in *in vitro* models, thus not integrating the complex surrounding environment of cells in vivo and the geometric constrain found in tissues. For instance, in skeletal muscles the dynamic of MT interactions are limited, here mitochondria appear globular and densely packed into the narrow space left by myofilaments, a condition that physically restrain MT dynamics. However, inhibition of fusion cause dysregulation of calcium oscillations during prolonged stimulation and impair excitation-contraction cycles, underlining the importance of MT fusion proteins in muscles, in vivo [121]. Furthermore, some discrepancies in the morphology of the mitochondria of the same cell lines, described by different laboratories, have been observed and may result from different culture conditions [122].

When there is limited damage, MT can also repair itself by eliminating damaged materials via mitochondrial-derived vesicles (MDVs) that bud off and then fuse with lysosomes [123]. More recently Hammerling and collaborators described another pathway for MT elimination, by which MT are sequestered into Rab5-positive early endosomes via the ESCRT-machinery (Endosomal Sorting Complexes Required for Transport) in a Parkin-dependent manner [124]. Although this endosomal pathway is activated by stress that also activates mitophagy, endosomal-mediated mitochondrial clearance seems to initiate before autophagy. Furthermore, for clearance of mitochondria by Parkin in *Atg5^−/−^* MEFs, DRP1-mediated mitochondrial fission is not required. From all these studies stem out that MT turnover is extremely complex and that on the base of the intensity of the stress and MT damage first MDVs are generated, later on endosomal-mediated mitochondrial clearance is induced and finally MT fission and then mitophagy may occur, thus mitophagy seems to represent rather the ultimate answer of cells in response to stress, probably right before cell death.

It was suggested that MT (especially ER/MT contact sites) supply membranes for autophagosomes biogenesis [125]. Later on, it emerged that the ER/MT relationship more generally controls autophagic process. Indeed tightening ER-MT contacts by overexpression of proteins involved in ER-MT contact sites such as VAPB or RMDN3 (Vesicle-associated membrane protein-Associated Protein B and Regulator of Microtubule Dynamics protein 3 respectively), impairs rapamycin- and Torin1-induced autophagy and not autophagy induced by starvation [126]. The mechanism by which the VAPB-RMDN3 tethers regulates autophagy seems to be related to their role in facilitating ER-MT Ca^2+^ exchanges but also correlating with MT dynamics [127]. 

One of the most complete molecular context describing the connection between MT morphology, ER-MT contacts and autophagy is probably the hypoxia induced mitophagy: in this context FUNDC1 integrates MT fission and mitophagy at the ER-MT contacts by working in concert with DRP1 and calnexin [128]. 

It stems out from several lines of evidence that a tight bidirectional regulation between the autophagic machinery and mitochondria morphodynamics exists. 

Interestingly enough, a function of ER-MT contacts in viral responses have been described [129] and as already said MAVS protein localizes at MAM. Autophagy is also known to play complex and unclear roles in virus replication [130]. It is thus possible to speculate that the profound relationship between MT and viral responses also involves autophagy. 

To further confirm the tight link between autophagy process and MT dynamics, in patients with dominant optic atrophy, as already said associated with Opa1 mutations, the severity of the symptoms seems to be controlled by autophagy [131]. 

Another example of this association is in hepatocellular carcinoma; here Ca^2+^ increase promotes MT fission by up-regulating expression of Drp1 and Fis1 [132]. Moreover, such feedback loop significantly promotes also autophagy by Ca^2+^/CAMKK/AMPK pathway (Calcium/calmodulin-dependent protein Kinase Kinase/AMP-activated protein kinase. This suggest that fission/fusion of MT, Ca^2+^ signalling and autophagy may work in concert, also during tumorigenesis. 

Finally, a profound relationship exists between neurological disorders and autophagy underlining once again that autophagy and MT shaping work together in similar pathological context. 

In these latest examples, the possible role of ER-MT contacts sites was not explored, it remains thus to be clarified if ER-MT contacts sites play role at the interface of the relationship between MT shape and autophagy in pathological context.

All these discoveries offered new lines of research and disclosed new targets for directed drug discovery. A better understanding of all these processes and all these intricate relationships likely will lead to a better understanding of the regulation of MT dynamics and a great improvement in human health.

## Figures and Tables

**Table 1:** Human disorders associated with disturbed mitochondrial dynamics.

Gene Carrying Mutations	Name of Pathology	Mitochondrial Defect	Refs.
OPA1 and OPA5	autosomal dominant optic atrophy	skin fibroblasts carrying Opa1 mutations show impaired oxidative phosphorylation and mitochondrial fusion	[110]
MFN2	Autosomal dominant Charcot-Marie-Tooth neuropathy type 2A	Alteration in mitochondrial fusion and trafficking along the axonal microtubule system	[111]
GDAP1	Charcot-Marie-Tooth Type 4A, recessively or dominantly inherited peripheral neuropathies	Defect in fission, fusion and transport of mitochondria. Fibroblasts from autosomal-recessive CMT4A patients display reduced mitochondrial membrane potential and reduced fission and GSH levels	[112]
DRP1	Abnormal brain development	fibroblasts obtained from the patient showed defective mitochondrial and peroxisomal fission	[113]
LETM1	The Wolf-Hirschhorn Syndrome, monoallelic deletion	Fragmentation of the mitochondrial network.	[114]
DNM1L		forming aggregates in the cytoplasm and on highly tubulated mitochondrial network, whereas neither structural difference of the peroxisome network, nor alteration of the respiratory machinery was noticed	[115,116]
SLC25A46	Ataxia, neurodegeneration	Impaired mitochondrial fusion	[117]
WBSCR16	Williams-Beuren syndrome, multigene deletion	Mice heterozygous for the mutation are shown to have neuronal mitochondria with reduced membrane potential and increased susceptibility to mitochondrial fragmentation in response to excitotoxic stress. Implications of the data are discussed	[118]

OPA: OPptic Atrophy 1; MFN: Mitofusin; GDAP1; DRP1: Dynamin-Related Protein 1; LETM1; DNM1L Dynamin 1 Like; SLC25A46; is a member of the mitochondrial solute carrier family SLC25; WBSCR16: Williams-Beuren Syndrome Chromosomal Region 16; CMT4A: Charcot-Marie-Tooth type 4A; GSH: glutathione

## Abbreviations

ADP	Adenosine DiPhosphate
AMP	Adenosine MonoPhosphate
AMPK	AMP-activated protein Kinase
ATP	Adenosin-TriPhosphate
Bcl-2	B-Cell Lymphoma 2
CAMKK	Calcium/calmodulin-dependent protein kinase kinase
CCCP	CarbonylCyanure m-ChloroPhénylhydrazone
CHO cells	Chinese hamster ovary cells
DRP1	Dynamin-Associated Protein 1
DRP2	Dynamin-Associated Protein 2
DsRNA	Double-Stranded RNA
ER	Endoplasmic Reticulum
ERK	Extracellular signal-regulated kinases
FIS1	mitochondrial FISsion 1 protein
FUNDC1	FUN14 Domain Containing 1
GDP	Guanosine-DiPhosphate
HIV	Human Immunodeficiency Virus
HBV	hepatitis B virus
HCV	hepatitis C virus
IFN-I	Type 1 interferon
LPS	Lipopolysaccharide
MAM	Mitochondria Associated-ER Membranes
MAVS	Mitochondrial Anti Viral-Signaling protein
MDA5	Melanoma Differentiation-Associated protein 5
MFF	Mitochondrial Fission Factor
Mfn	Mitofusin
MiD49/51	Mitochondrial Dynamics protein 49/51
MIM	Mitochondrial Inner Membrane
Miro1/2	Mitochondrial Rho GTPase1/2
MOM	Mitochondrial Outer Membrane
MT	Mitochondria
mTOR	Mammalian/Mechanistic Target of Rapamycin
NDV	Newcastle Disease Virus
NF- κB	Nuclear Factor κB
NLRP3	NOD-Like Receptor family, Pyrin domain containing 3
OMA1	metallopeptidase Overlapping with the *m*-AAA protease
OPA1	OPtic Atrophy type 1
Pink1	PTEN-induced putative kinase 1
PKA	Protein Kinase A
RIG1	Retinoic acid-Inducible gene I
RMDN3	Regulator of Microtubule Dynamics protein 3
ROS	Reactive Oxygen Species
SIMH	Stress-Induced Mitochondrial Hyperfusion
Sirt5	Sirtuin5
SLP2	Stomatin Like Protein 2
VAPB	Vesicle-Associated membrane Protein-associated Protein B
VopE	*Vibrio cholerae* Type 3 secretion system effector
YME1L	Yeast Mitochondrial Escape protein 1
